# Outcome of E1224-Benznidazole Combination Treatment for Infection with a Multidrug-Resistant Trypanosoma cruzi Strain in Mice

**DOI:** 10.1128/AAC.00401-18

**Published:** 2018-05-25

**Authors:** Lívia de Figueiredo Diniz, Ana Lia Mazzeti, Ivo Santana Caldas, Isabela Ribeiro, Maria Terezinha Bahia

**Affiliations:** a Laboratório de Doenças Parasitárias, Escola de Medicina & Núcleo de Pesquisas em Ciências Biológicas, Universidade Federal de Ouro Preto, Minas Gerais, Brazil; b Laboratório de Parasitologia Básica, Instituto de Ciências Biomédicas, Universidade Federal de Alfenas, Alfenas, Minas Gerais, Brazil; c Drugs for Neglected Disease *initiative* (DND*i*), Geneva, Switzerland

**Keywords:** Trypanosoma cruzi, combination therapy, E1224, benznidazole combination

## Abstract

Combination therapy has been proposed as an alternative therapeutic approach for the treatment of Chagas disease. In this study, we evaluated the effect of treatment with benznidazole combined with E1224 (ravuconazole prodrug) in an experimental murine model of acute infection. The first set of experiments assessed the range of E1224 doses required to induce parasitological cure using Trypanosoma cruzi strains with different susceptibilities to benznidazole (Y and Colombian). All E1224 doses were effective in suppressing the parasitemia and preventing death; however, parasitological cure was observed only in mice infected with Y strain. Considering these results, we evaluated the effect of combined treatment against Colombian, a multidrug-resistant T. cruzi strain. After exclusion of antagonistic effects using *in vitro* assays, infected mice were treated with E1224 and benznidazole in monotherapy or in combination at day 4 or 10 postinoculation. All treatments were well tolerated and effective in suppressing parasitemia; however, parasitological and PCR assays indicated no cure among mice treated with monotherapies. Intriguingly, the outcome of combination therapy was dependent on treatment onset. Early treatment using optimal doses of E1224-benznidazole induced a 100% cure rate, but this association could not eliminate a well-established infection. The beneficial effect of combination therapy was evidenced by further reductions of the patent parasitemia period in the group receiving combined therapy compared with monotherapies. Our results demonstrated a positive interaction between E1224 and benznidazole against murine T. cruzi infection using a multidrug-resistant strain and highlighted the importance of a stringent experimental model in the evaluation of new therapies.

## INTRODUCTION

Chagas disease is a parasitic infection caused by the protozoan Trypanosoma cruzi, which was discovered in 1909 by Carlos Chagas. This infection is recognized by the World Health Organization as one of the world's 20 most neglected tropical diseases and is responsible for substantial morbidity and mortality, particularly in the poorer areas of Latin America (1). Although the disease has been identified and described for more than 100 years, only two therapeutic alternatives are available: benznidazole and nifurtimox, medicines that are known to cause serious toxicity and requiring extended treatment courses with unsatisfactory cure rates, especially when used in the chronic phase of the infection.

Novel antifungal triazole derivatives that inhibit ergosterol biosynthesis have arisen as alternative treatments for Chagas disease. T. cruzi depends exclusively on endogenously produced sterol for growth and survival, and some triazole derivatives exhibit pharmacokinetic properties suitable for the treatment of this disseminated intracellular infection (2). A number of ergosterol inhibitors have been tested in animal models of Chagas disease, including posaconazole, ravuconazole, and VNI (3–7). Posaconazole induced a curative effect in both the acute and chronic phases of experimental Chagas disease (3, 4). However, the disadvantage of posaconazole is the complexity and cost of manufacturing this drug (8). The novel CYP51 inhibitor VNI, the first nonantifungal compound to target the 14α-demethylase activity of T. cruzi, rather than an antifungal program, was proven to cure both the acute and chronic phases of infection with the Tulahuen T. cruzi strain in mice (6). However, this compound failed to cure mice infected with the Y and Colombian T. cruzi strains in both phases of the infection (7). Of particular interest, ravuconazole has previously been shown to exhibit potent *in vitro* activity, although its *in vivo* action in murine models of acute and chronic Chagas disease is limited (10). Similarly, this drug has been shown to exhibit potent but not curative activity in a dog model of Chagas disease (5). The lack of a curative effect of ravuconazole in these animal models is probably due to the short half-life of the drug in both mice and dogs (5, 10). However, due to its poor solubility, the compound was not suitable for use as an oral or injectable drug, which was the original development goal. In 2009, the Drugs for Neglected Diseases *initiative* (DND*i*) joined forces with Eisai Co. Ltd., the Japanese pharmaceutical company that discovered ravuconazole, to develop E1224 as a new chemical entity for Chagas disease. E1224 is a prodrug that is readily converted to ravuconazole, which improves drug absorption and bioavailability (11).

The results of phase II clinical trials to evaluate the efficacy and safety of posaconazole and E1224 have recently been disclosed. These clinical trials indicated the safety of both drug candidates in humans, but unfortunately, they exhibited little to no sustained efficacy in treating patients in the chronic phase of Chagas disease when administered as monotherapy (11, 12). On the other hand, Pinazo et al. (13) described the successful resolution of a T. cruzi infection following treatment with posaconazole in a prolonged therapeutic scheme; benznidazole had previously reduced but not completely eliminated parasitemia in this patient. These findings highlight the need to investigate alternative dosing regimens and possible combination therapies to improve treatment efficacy and safety.

Given the aforementioned findings, the aims of this study were the following: (i) evaluate the *in vitro* activity of ravuconazole in combination with benznidazole using the H9c2 rat cardiomyoblast line infected with the Y and Colombian T. cruzi strains; (ii) evaluate the *in vivo* activity of E1224 in mice infected with T. cruzi strains with differing levels of benznidazole susceptibility, using state-of-the-art methods to demonstrate cure; and (iii) investigate the anti-T. cruzi efficacy of E1224 in combination with benznidazole in an experimental murine model of acute Chagas disease to support the potential clinical evaluation of such combination therapies.

## RESULTS

To assess the range of E1224 doses that can cure T. cruzi infection in mice, animals infected with the Y strain were treated with 10, 20, 30, 40, and 50 mg/kg of body weight of E1224 per day for 20 days. The times required for E1224 to suppress the parasite were similar among all evaluated doses and comparable to that of the standard benznidazole treatment (Table 1). Of mice treated with E1224, 71.5% (20 mg/kg and 40 mg/kg), 85.7% (30 mg/kg and 50 mg/kg) and 100% (10 mg/kg) were cured at the end of a 6-month follow-up period, versus 87.5% for benznidazole at 100 mg/kg (Table 1). Interestingly, all evaluated doses of E1224 induced similar cure rates among animals infected with the T. cruzi Y strain.

**TABLE 1:** Efficacy of E1224 treatment for 20 days in a Trypanosoma cruzi murine model^*a*^

Exptl group^*b*^	No. of surviving mice/total	No. of mice with negative FBE^*c*^/total	No. of mice with negative blood PCR^*d*^ results/total	No. of mice with negative results/total (%)
E1224				
10 mg/kg	7/7	7/7	7/7	7/7 (100)
20 mg/kg	7/7	6/7	5/6	5/7 (71.5)
30 mg/kg	7/7	7/7	5/7	6/7 (85.7)
40 mg/kg	7/7	7/7	6/7	5/7 (71.5)
50 mg/kg	7/7	6/7	5/6	6/7 (85.7)
Bz,^*f*^ 100 mg/kg	7/7	6/7	5/6	6/7 (85.7)
Untreated	0/7	0/7	ND^*e*^	0/7 (0)
Uninfected	7/7	7/7	7/7	7/7 (100)

a Swiss female mice (7/group) weighing 20 to 24 g were inoculated with 5 × 10^3^ trypomastigotes (Y strain).

b Oral treatment was initiated on the 4th day after inoculation and continued for 20 days.

c FBE, fresh blood examination performed before and after cyclophosphamide immunosuppression.

d PCR assay was performed at the 1st and 6th months after treatment.

e ND, not detected (all mice died before the 30th day of infection).

f Bz, benznidazole.

All treatment regimens were effective in preventing death. The drug was well tolerated by animals, and no mortality was detected among animals receiving these treatments. Additionally, weight gain did not differ between treated and uninfected animals during all evaluated periods (data not shown).

**FIG 1:**
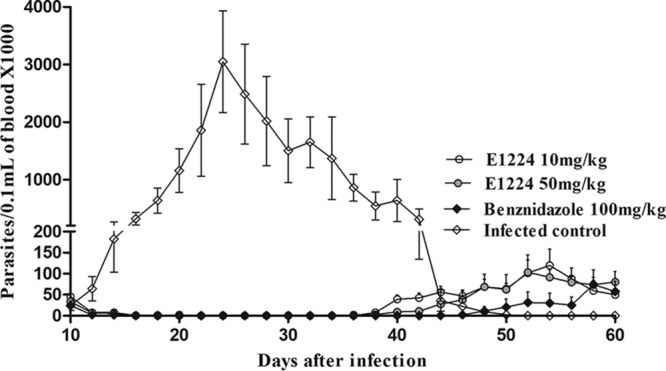
Parasitemia levels after oral administration of E1224 or benznidazole. Shown is the parasitemia curve obtained for mice infected with 5,000 trypomastigotes of T. cruzi Colombian strain treated daily with 10 and 50 mg/kg of E1224 and 100 mg/kg of benznidazole for 20 consecutive days. Treatments were started at day 10 of infection.

Given the high efficacy of E1224 against the T. cruzi Y strain, further experiments were performed using the benznidazole-resistant Colombian strain (14). Consistently, all treatments cleared parasitemia during the treatment period (Fig. 1), but parasitemia clearance was faster in animals treated with 50 mg/kg of E1224 and 100 mg/kg of benznidazole (approximately 5 treatment days) than in animals treated with 10 mg/kg of E1224 (11.6 days [Table 2]). Despite the treatment, parasite rebound was detected after the end of treatment with both E1224 and benznidazole in monotherapy. Our findings showed neither E1224 nor benznidazole was able to induce parasitological cure in mice infected with the Colombian strain when administered for 20 days (Fig. 1 and Table 2), as evidenced by parasitemia reactivation and positive quantitative PCR (qPCR) results for all treated mice. The same results were obtained using mice infected with the VL-10 strain, which is also highly resistant to benznidazole (data not shown). However, whereas untreated animals infected with the Colombian strain succumbed (75%) to the infection, all treatments were effective in preventing death.

**TABLE 2:** Effect of E1224 or benznidazole treatment on the benznidazole-resistant Colombian Trypanosoma cruzi strain^*a*^

Exptl group^*b*^	No. of mice that died/total	No. of mice showing parasitemia clearance/total	No. of doses achieving parasitemia clearance	No. of mice with positive FBE or PCR result/total^*c*^
E1224				
10 mg/kg	0/7	7/7	11.6 ± 4.0	7/7
50 mg/kg	0/7	7/7	5.8 ± 2.03	7/7
Bz, 100 mg/kg	0/7	7/7	5.4 ± 2.8	7/7
Infected control	5/7	0/7		7/7
Uninfected	0/7			

a Swiss female mice (7 to 10/group), weighing 20 to 24 g, were inoculated with 5 × 10^3^ trypomastigotes of the Colombian strain.

b Oral treatment was initiated 10 days after inoculation and continued for 20 days.

c Parasitemia was followed for 60 days after treatment; PCR assay was performed during the 1st and 6th months after treatment.

As treatment with E1224 effectively suppressed parasitemia and prevented death in animals infected with strains of the parasite that are resistant to the reference drug, we evaluated the effects of the drugs in combination. To exclude antagonistic effects, the nature of the interaction between benznidazole and ravuconazole against amastigote forms of the Y and Colombian strains was evaluated in H9c2 rat cardiomyoblasts.

**FIG 2:**
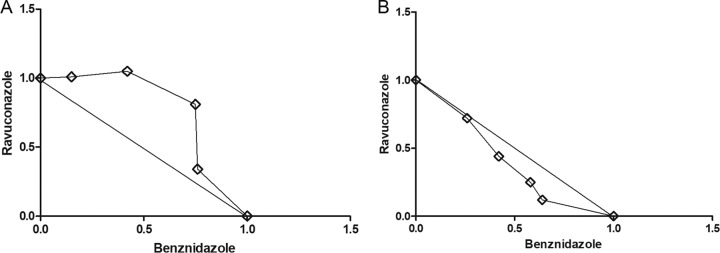
Representative isobolograms for *in vitro* interactions between benznidazole and ravuconazole against Colombian (A) and Y (B) Trypanosoma cruzi strains. Interactions are given at the EC_50_ level. Numbers on the axes represent normalized FICs of benznidazole (*x* axis) and ravuconazole (*y* axis).

The benznidazole-ravuconazole interaction *in vitro* was assessed using a modified fixed-ratio isobologram method, and the data were analyzed at the 50% effective concentration (EC_50_) level (15). The mean sums of fractional inhibitory concentrations (∑FICs) of two independent experiments are presented in Table 3. Representative isobolograms are shown in Fig. 2. Interactions were classified as described by Seifert and Croft (16) as follows: synergistic, mean ∑FIC of ≤0.5; antagonistic, mean >4.0; and indifferent, mean ∑FIC between 0.5 and 4.0. The interaction of benznidazole with ravuconazole was classified as indifferent, i.e., a simple additive effect, based on the mean ∑FICs of 0.76 to 0.98 for the Y strain and 1.10 to 1.56 for the Colombian strain at the four drug ratios (1:4, 2:3, 3:2, and 4:1) tested (Fig. 2 and Table 3).

**TABLE 3:** Mean ∑FICs of the interaction between ravuconazole and benznidazole toward intracellular amastigotes of the Y and Colombian Trypanosoma cruzi strains^*a*^

Bz/Rav ratio	Y strain			Colombian strain		
	Bz FIC	Rav FIC	∑FIC	Bz FIC	Rav FIC	∑FIC
4:1	0.64 ± 0.004	0.12 ± 0.12	0.76	0.76 ± 0.19	0.34 ± 0.15	1.10
3:2	0.58 ± 0.26	0.25 ± 0.15	0.83	0.75 ± 0.04	0.81 ± 0.37	1.56
2:3	0.42 ± 0.16	0.44 ± 0.30	0.86	0.42 ± 0.25	1.05 ± 0.09	1.45
1:4	0.26 ± 0.18	0.72 ± 0.035	0.98	0.15 ± 0.07	1.01 ± 0.26	1.16
Mean FIC in combination	0.47 ± 0.15	0.39 ± 0.15	0.86	0.52 ± 0.29	0.79 ± 0.32	1.31

a FICs, fractional inhibitory concentrations at the IC_50_ level; Bz, benznidazole; Rav, ravuconazole.

Considering the additive effect of ravuconazole and benznidazole observed *in vitro*, we assessed whether E1224 administered in combination with benznidazole is more effective than each drug alone in treating mice infected with the benznidazole-resistant Colombian strain of T. cruzi. Due to the high intrinsic resistance of this strain, higher doses of E1224 were used in this experiment (37.5 and 50 mg/kg twice a day [b.i.d.] every 12 h [q12h]) in combination with 75 and 100 mg/kg of benznidazole. The drugs were administered at two different time points for 20 consecutive days: on day 4 of infection and after parasitemia detection in the peripheral blood (10 days postinoculation [Table 4]). Among animals treated on day 4 of infection, both E1224 and benznidazole were unable to cure mice infected with the Colombian strain when administered as monotherapy for 20 days (Table 4). Parasitemia rebound was detected after the end of treatment in 100% and 75% of mice treated with E1224 and benznidazole, respectively. Conversely, parasitemia rebound was detected in only 50% of animals that received 37.5 mg/kg of E1224 in combination with 50 mg/kg of benznidazole (Table 4). In addition, 100% of animals that received 50 mg/kg of E1224 in combination with 100 mg/kg of benznidazole exhibited negative fresh blood examination and PCR results. Consistently, all treatments reduced parasitemia levels in infected and treated animals (*P* < 0.05), but the parasitemia levels detected in the peripheral blood of animals receiving combined treatments were significantly lower than those in animals treated with each drug alone (*P* < 0.05) (Fig. S1). The results emphasize that E1224 and benznidazole combination therapy is more effective in reducing circulating parasite levels and inducing parasitological cure than either of the drugs given alone, even against drug-resistant organisms.

**TABLE 4:** Efficacy of E1224 in monotherapy or combined with benznidazole for 20 days in a Trypanosoma cruzi drug-resistant murine model^*a*^

Time point	Exptl group^*b*^	Parasitemia clearance, days (mean ± SD)	Parasitemia relapse, days (mean ± SD)	Patent parasitemia, days (mean ± SD)	Parasitemia AUC±SD (×10^3^)^*c*^	No. of surviving/total (%)^*d*^	Total negative assays/total (%)^*e*^
4th dpi	E1224 (50)	1.22 ± 0.66	28.85 ± 3.98	29.10 ± 3.98	1,859 ± 1,345	9/9 (100)	0/9 (0)
	Bz (100)	1	24.16 ± 10.88	22.16 ± 9.43	814 ± 1026	8/8 (100)	2/8 (25)
	E1224 (50) + Bz (100)	1	ND	ND	0	10/10 (100)	10/10 (100)
	E1224 (37.5)	1.22 ± 0.66	18.4 ± 3.75	26 ± 3.53	2,378 ± 3,452	7/7 (100)	0/7 (0)
	Bz (75)	1	18.33 ± 3.51	28 ± 3.60	489 ± 755	7/7 (100)	0/7 (0)
	E1224 (37.5) + Bz (75)	1	37.75 ± 7.36	13.25 ± 0.27	54 ± 124	10/10 (100)	4/10 (40)
10th dpi	E1224 (50)	5.85 ± 2.03	8.71 ± 3.30	36.71 ± 2.28	1,513 ± 869	7/7 (100)	0/7 (0)
	Bz (100)	5.42 ± 2.87	28.14 ± 11.35	26.28 ± 7.52	530 ± 863	7/7 (100)	0/7 (0)
	E1224 (50) + Bz (100)	4.14 ± 2.41	27.57 ± 3.04	15.85 ± 5.52	53 ± 32	7/7 (100)	0/7 (0)
	E1224 (37.5)	6,66 ± 1.96	10.28 ± 1.11	36 ± 5.06	1,031 ± 622	7/7 (100)	0/7 (0)
	Bz (75)	5.57 ± 1.61	22.85 ± 4.52	24.14 ± 4.45	328 ± 525	7/7 (100)	0/7 (0)
	E1224 (37.5) + Bz (75)	4.0 ± 1.73	28.57 ± 4.85	16.71 ± 5.46	84 ± 101	7/7 (100)	0/7 (0)
	Infected untreated	43 ± 5.57	ND	43 ± 5.57	23,836 ± 12,317	4/15 (26.6)	0/15 (0)

a Swiss female mice (7 to 10/group) weighing 20 to 24 g were inoculated with 5 × 10^3^ trypomastigotes of the benznidazole-resistant Colombian strain.

b Oral treatment was initiated on the 4th or 10th day postinoculation (dpi) and continued for 20 days. Numbers in parentheses are doses in milligrams per kilogram.

c Area under the curve of parasitemia until 60 days after infection.

d Survival until 30 days after treatment.

e Negative fresh blood examination and negative PCR assay results performed during the 1st and 6th months after treatment.

When benznidazole and E1224 were used to treat an established infection with the Colombian strain for 20 days starting 10 days after inoculation, the drugs were unable to induce parasitological cure, alone or in combination (Table 4), as evidenced by identical levels of parasitemia reactivation in all mice after the end of treatment. However, although the combined treatment did not cure mice when administered 10 days after infection, it efficiently reduced the patent parasitemia period (Table 4). In an attempt to quantify these effects, the percent reduction in the patent parasitemia period was calculated for each treated group and compared with those calculated for the untreated controls. Whereas the period of patent parasitemia was reduced by 3.4% in the group treated with 50 mg/kg of E1224 and by 31% in the group treated with 100 mg/kg of benznidazole, a 58% reduction was observed in the group receiving combined therapy. Likewise, when analyzing the area under parasitemia curve until 60 days after treatment, it was observed that mice treated with combined drugs showed a significant reduction in parasite load in the blood in comparison with animals treated with monotherapies in the same period (Table 4).

**FIG 3:**
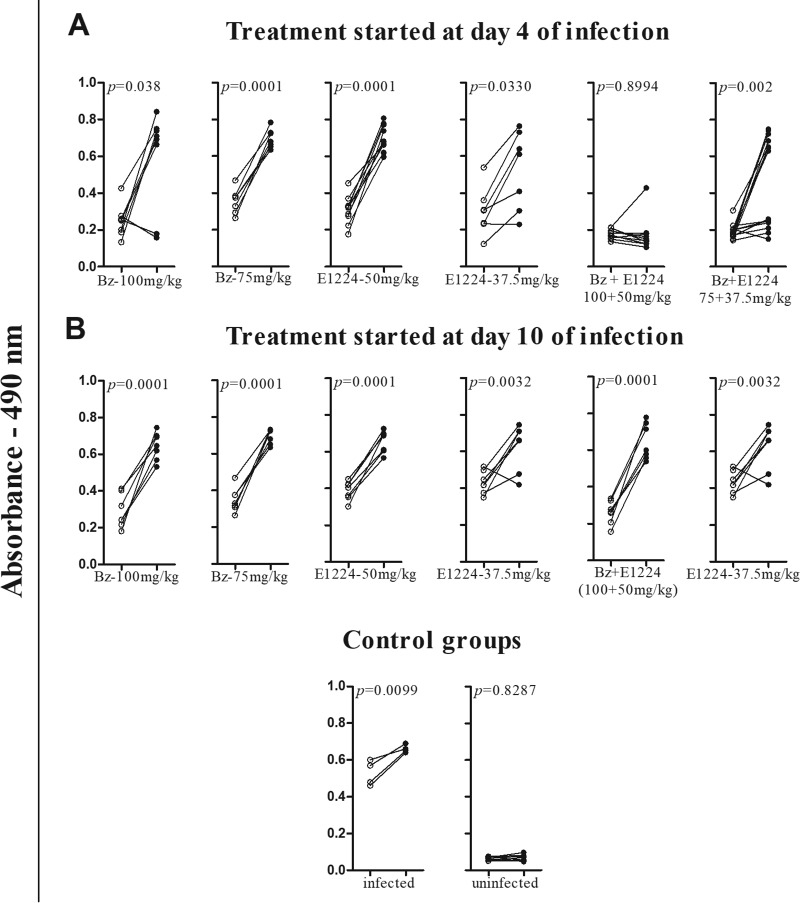
Effects of benznidazole and E1224 treatments alone or in combination on IgG antibody level. IgG antibodies in sera of mice infected with 5,000 trypomastigotes of the T. cruzi Colombian strain and treated daily with 37.5 and 50 mg/kg/day of E1224 in combination with 50 and 100 mg/kg of benznidazole for 20 consecutive days were measured. Treatments were started at day 4 (A) or 10 (B) of infection. White circles represent the IgG levels 30 days after treatment, and black circles represent IgG levels 180 days after treatment.

Figure 3 presents the IgG levels measured at 30 and 180 days after treatment with E1224 and benznidazole alone or in combination. Figure 3A shows the IgG levels in the sera of animals receiving drug treatment 4 days after inoculation. A significant increase in the IgG levels was detected in all infected and untreated animals. The same results were obtained for most animals that received benznidazole and E1224 monotherapy. In contrast, antibody levels remained stable in 90% of animals treated with higher doses and in 50% of those treated with lower doses of the drug combination. In these animals, the antibody levels were similar to those detected in the serum of healthy mice. In contrast, sera obtained from animals treated starting on the 10th day of infection exhibited an increase in the anti-T. cruzi antibody levels in all study groups (Fig. 3B). These results are in agreement with the parasitological evaluation because the parasite and/or its DNA was detected in the sera of all treated mice.

## DISCUSSION

Different therapeutic strategies have been explored in the path toward new treatments for Chagas disease, including alternative doses and treatment duration of current drugs, combination therapy, and drug repositioning, which is the use of an existing drug for a new application. Drug repositioning has been successfully applied to different diseases (17) but has yet to bear fruit for Chagas disease. A number of studies suggested that CYP51 inhibitors are the most efficacious class of drugs in animal models of Chagas disease (18). More recent *in vitro* and *in vivo* information suggested variable response rates with well-known antifungal agents (19, 20). Notably, this class of compounds did not show the same efficacy in the treatment of human disease, highlighting the need to investigate alternative dosing regimens and possible therapeutic combinations for improving the effectiveness of the treatment. Drug associations can reduce parasite load, decrease mortality rates, and reduce tissue lesions caused by the parasite. In addition, since one of the key limitations of benznidazole relates to safety, tolerability, and the long duration of treatment, alternative treatment regimens could potentially impact overall treatment compliance. This study was designed to evaluate the activity of E1224 in combination with benznidazole in order to improve the treatment of Chagas disease.

The range of E1224 doses that cure T. cruzi infection in mice was established prior to the evaluation of the combination treatment. Our findings showed that E1224 treatment yields a high cure rate in animals infected with the T. cruzi Y strain over a wide range of concentrations (10 to 50 mg/kg). All evaluated doses were able to induce similar levels of cure, demonstrating that this T. cruzi strain is highly susceptible to the evaluated compound. Based on these results, the compound was evaluated in a more stringent model of drug resistance. Animals infected with the Colombian strain were treated with lower and higher doses of E1224 than those previously examined. In this model that involves highly resistant benznidazole strains, E1224 was able to suppress but not cure parasitemia in infected animals. Specifically, parasitemia relapse was detected in all treated animals, irrespective of the drug dose. These observations corroborate those reported by others (5, 7, 8, 10, 21–23), that the response of an experimental T. cruzi infection to treatment with CYP51 inhibitors differs significantly depending on parasite strain. A number of previous studies have shown that some T. cruzi strains are multidrug resistant (7, 14, 18), such as VL-10, which is resistant to benznidazole, posaconazole (8, 23), and E1224 (data not shown). Other studies have verified that T. cruzi strains susceptible to benznidazole are resistant to CYP51 inhibitors (5, 21, 22), suggesting that both multidrug resistance and selective drug resistance are possible among T. cruzi strains. Although the molecular basis of the differential drug resistance remains poorly understood, these findings emphasize the importance of exploring the potential of E1224 and benznidazole in combination for the specific treatment of Chagas disease.

First, the nature of the interaction between ravuconazole and benznidazole was determined *in vitro* against T. cruzi strains exhibiting different degrees of benznidazole resistance. The drug combinations resulted in a positive effect, since all ∑FICs fell in the “additivity range” (0.5 < FIC < 4), regardless of the parasite strain used. Although a synergistic effect would be more desirable, the additive effect, also called an indifferent interaction, can be considered a positive outcome, since *in vivo* biological interactions can contribute to a favorable combined effect (24, 25). In this way, the *in vitro* results indicate a potential beneficial effect of the benznidazole-ravuconazole combination and justify its evaluation *in vivo* using mice as an experimental model. Although the same effect was verified for all benznidazole-ravuconazole ratios evaluated, a 1:1 ratio of E1224 to benznidazole was selected for the *in vivo* experiments.

The combination of benznidazole with inhibitors of the sterol C14α demethylase consistently showed a beneficial effect in experimental studies. Recently, the combination of posaconazole with benznidazole was demonstrated to be significantly more effective than single-dose regimens in terms of reducing the time of treatment (26) or drug dose (8) to cure mice infected with T. cruzi strain Tulahuen or Y. The ability of inhibitors of TcCYP51 to alter the pharmacokinetic profile of benznidazole, as reported for itraconazole (27), with an increase of 2.7-fold in the volume of distribution and 7.5-fold in the elimination half-life documented in coadministration, may be one of the factors responsible for the enhanced efficacy of these combinations. The use of different combination therapy protocols for azoles and benznidazole is generally reported to be beneficial, but this strategy has not been evaluated in mice infected with T. cruzi strains resistant to both drugs, such as the Colombian strain. Our data extend previously reported data regarding the effect of sterol C14α demethylase inhibitors and benznidazole on T. cruzi infection.

Our findings demonstrate that the E1224-benznidazole combination improves the treatment response of drug-resistant T. cruzi infection in mice, but this improvement is clearly related to the length of infection. The combination of benznidazole and E1224 effectively cured acute infections of the drug-resistant Colombian strain in mice when administered early in the course of infection, but the same effect was not observed in animals treated with monotherapy. All animals that received the higher dose and 40% of those treated with lower doses of the drug combination had negative results in fresh blood examination and qPCR, as well as significantly decreased IgG antibody levels in serum samples collected 6 months after treatment. However, among the animals treated with the same doses in monotherapy, negative qPCR assay results and decreased antibody levels were obtained for only two animals treated with 100 mg/kg of benznidazole.

When the mice infected with the drug-resistant strain were treated starting 10 days after infection, we did not observe the same effect as for early treatment. Both E1224 and benznidazole, in monotherapy or combination, suppressed but did not cure the infection. In this case, only a reduction of circulating parasite levels in blood of mice treated with combinations was observed. Others have reported that more than 50% of mice infected with the drug-resistant strain were cured early in the course of infection, but few were cured once the infection had been well established (28). One may hypothesize that the established infection could require longer exposure to the drug combination, as in other conditions. Urbina (35, 36) discusses whether the therapeutic failure observed in recent clinical trials against chronic Chagas disease may be related to suboptimal treatment durations.

The differences in drug efficacy in the treatment of early or established T. cruzi infections caused by the same strain suggest that different patterns of response to treatment may be related to the tissue distribution of parasites (time of infection), as well as the drug concentration in different host tissues, not genetic resistance, reinforcing the hypothesis of other investigators (28). In line with this idea, our data raise important questions for preclinical studies of new drugs or therapeutic strategies for Chagas disease, related to parasite strains, timing of initiation of treatment, and assessment of drug exposure.

### Conclusion.

Our data show that combination therapy with E1224 and benznidazole is more effective in reducing circulating parasite levels than monotherapies in acute T. cruzi infection caused by drug-resistant organisms; however, this therapeutic scheme induced parasitological cure only when applied early in the course of infection. Finally, this work reinforces the importance of evaluating and standardizing preclinical models in drug efficacy studies, including the use of different parasite strains and infection times prior to treatment.

## MATERIALS AND METHODS

### Parasites.

Trypanosoma (Schizotrypanum) *cruzi* strains Colombian (DTU I) and Y (DTU II) (29) were used in this study. The Y strain is partially resistant and the Colombian strain is highly resistant to benznidazole (30).

### Mammalian cell cultures.

H9c2, an embryonic rat ventricular cell line, was used for both drug toxicity and infection assays. The cultures were sustained in Dulbecco's modified Eagle's medium (DMEM) supplemented with 10% fetal bovine serum (FBS), 1 mM l-glutamine, and 100 μg/ml of penicillin-streptomycin (31). Cell cultures were maintained at 37°C in an atmosphere of 5% CO_2_ and air, and assays were conducted at least three times in duplicate.

### Compounds. (i) Test compounds.

Ravuconazole [(*R*-(R*,R*))-4-(2-(2-(2,4-difluorophenyil)-2-hydroxy-1-metlyl-3-(1H-1,2,4-triazol-1-yl)propyl)-4-thiazolyl)benzonitrile; Eisai, Japan] was used *in vitro*. E1224, dihydrogen phosphonoxy methoxy-derived ravuconazole, which is a prodrug of ravuconazole (Eisai, Japan), was used *in vivo*.

### (ii) Reference compound.

Benznidazole [2-nitroimidazole-*N*-benzyl-2-nitro-1-imidazole acetamide; Lafepe] was used as the reference treatment.

For *in vitro* studies, stock solutions of ravuconazole (1 mM) and benznidazole (100 mM) were prepared in dimethyl sulfoxide (DMSO). All subsequent dilutions were prepared in the respective fresh culture medium (DMEM) on the day of the assay. The final DMSO concentration never exceeded 0.6%; this concentration is not toxic to parasites or mammalian cells. For *in vivo* studies, E1224 was solubilized in distilled water, and benznidazole was administered in aqueous suspensions containing 0.5% (wt/vol) methyl cellulose.

### Determination of ravuconazole and benznidazole interactions against the amastigote forms of the Y and Colombian T. cruzi strains.

The *in vitro* assays were carried out using the H9c2 rat cardiomyoblast line infected with highly invasive trypomastigote forms of the Y and Colombian T. cruzi strains, which have been shown to infect at least 50% of exposed H9c2 cells. The drug interactions were assessed using a fixed-ratio method (15). Host cells were dispensed into 24-well tissue culture plates, containing coverslips, at 1 × 10^4^/well. After 24 h, the cells were infected with trypomastigotes obtained from tissue culture at a ratio of 10 parasites per cell (incubation for 24 h). Predetermined 50% effective concentration (EC_50_) values were used to determine the maximum concentrations of the individual drugs to ensure that the EC_50_ fell near the midpoint of a six-point 2-fold dilution series. The maximum concentrations used were 20 μM for benznidazole and 24 nM for ravuconazole. These concentrations were used to prepare fixed-ratio solutions at ratios of 5:0, 4:1, 3:2, 2:3, 1:4, and 0:5 of benznidazole to ravuconazole. All tissue culture slides were maintained at 37°C in a 5% CO_2_-air mixture. After 72 h, the cultures were fixed with methanol, stained with Giemsa, and microscopically examined to determine the percentage of cells infected in treated and untreated controls.

### *In vivo* efficacy studies.

Female Swiss mice from the Animal Facility at Ouro Preto Federal University (UFOP) in Minas Gerais State, Brazil, were used in this study. The animals were housed at a maximum of 7 per cage in a conventional room at 20 to 24°C under a 12/12-h light/dark cycle. The mice were supplied with a commercial feed and water that were available *ad libitum*. Swiss mice (18 to 23 g) were inoculated intraperitoneally with 5.0 × 10^3^ bloodstream trypomastigotes of the Y or Colombian T. cruzi strain.

The first set of experiments was designed to determine the efficacy of E1224 to cure mice infected with the Y strain. These mice were treated with 5 different doses of E1224: 10, 20, 30, 40, and 50 mg/kg of drug b.i.d. (q12h). The drug was administered at the time of parasitemia detection (4 days postinoculation) for 20 consecutive days. The parasitological cure rate was determined as described below, and the results were compared to those achieved using the reference treatment, benznidazole at 100 mg/kg. A group of animals infected with the parasite but receiving no treatment was used as the control.

The second set of experiments was designed to determine the efficacy of E1224 to induce parasitological cure in mice infected with the Colombian strain, which is classified as highly resistant to benznidazole. Groups of infected mice were treated with 10 or 50 mg/kg of E1224 b.i.d. (q12h) for 20 consecutive days. Treatments were started 10 days after inoculation, which is the time of parasitemia detection for the Colombian strain. The results were compared to those obtained for mice treated with 100 mg/kg of benznidazole and with those for untreated control mice, which were either infected with parasites or left uninfected.

We also assessed whether E1224 administered in combination with benznidazole would be more effective than each drug alone in treating mice infected with the benznidazole-resistant Colombian T. cruzi strain. Due to the high drug resistance profile of the Colombian strain, higher doses of the drugs were used, both alone and in combination. Thus, animals were treated with 37.5 and 50 mg/kg of E1224 b.i.d. (q12h) combined with 75 and 100 mg/kg of benznidazole. The drugs were administered for 20 consecutive days starting at two different times: (i) on day 4 of infection and (ii) at the time of parasitemia detection in the peripheral blood, i.e., 10 days postinoculation. The results were compared to those obtained with benznidazole or E1224 treatment administered alone at the same doses as used in combined treatment and with the control groups, which were either infected or uninfected.

### Assessment of parasitological cure.

Cure was determined following the methodology standardized by Caldas et al. (32), which is based on two parasitological methods: fresh blood examination before and after cyclophosphamide immunosuppression (CyI), followed by PCR assays performed on blood samples from mice negative for parasitemia. Animals presenting negative results for all tests were considered cured.

### Parasitemia.

To determine the natural reactivation of infection, the animals' parasitemia was evaluated for up to 30 days posttreatment by fresh blood examination. Five microliters of blood collected from the tail vein was examined and the parasite number was estimated as described previously (37). Animals with negative results in the parasitological tests were submitted to CyI, which consisted of three cycles of 50 mg of cyclophosphamide/kg of body weight for four consecutive days, with 3-day intervals between cycles. Parasitemia relapse was then followed during the CyI cycles and for 10 days thereafter.

### Real-time PCR.

Genomic DNA was purified from the collected blood 30 and 180 days posttreatment. DNA extraction was performed using the Wizard Genomic DNA purification kit (Promega), with some modifications (33). qPCRs were performed to amplify T. cruzi DNA using the SYBR green system (Roche Applied Science, Mannheim, Germany) according to the manufacturer's instructions and using the primers TCZ-F (5′-GCTCTTGCCCACAMGGGTGC-3′, where M = A or C) and TCZ R (5′-CCAAGCAGCGGATAGTTCAGG-3′) (Invitrogen) (34). The internal control, a segment of the murine tumor necrosis factor alpha (TNF-α) gene, was amplified using the primers TNF-5241 (5′-TCCCTCTCATCAGTTCTATGGCCCA-3′) and TNF-5411 (5′-CAGCAAGCATCTATGCACTTAGACCCC-3′) (Invitrogen) (34). Cycles of amplification were carried out in an ABI 7300 real-time PCR system from Applied Biosystems. The cycles consisted of an initial denaturation hold of 10 min at 95°C followed by 40 cycles of 15 s at 94°C and 1 min at 64.3°C with fluorescence acquisition. Amplification was immediately followed by a melt program with an initial denaturation for 15 s at 95°C, cooling to 60°C for 1 min, and then a stepwise temperature increase of 0.3°C/s from 60 to 95°C. All samples were analyzed in duplicate, and negative samples and reagent controls were processed in parallel for each assay.

### Influence of the specific treatment on IgG antibody profile.

T. cruzi-specific antibodies were used as reported by Caldas et al. (32). Briefly, enzyme-linked immunosorbent assay plates were coated with T. cruzi antigen alkaline extracted from strain Y during the exponential growth phase in liver infusion tryptose (LIT) medium. Anti-mouse IgG peroxidase-conjugated antibody (Sigma Chemical Co.) was used as the secondary antibody. The mean absorbance for 10 negative-control samples was used to determine the reactivity index value, which was obtained by dividing the absorption value (optical density [OD] value) of each serum sample by the mean value of the differential control sample.

### Statistical analysis: FIC index, isobologram construction, and classification of the nature of the *in vitro* interaction.

The fractional inhibitory concentrations (FICs) and the sums of FICs (∑FICs) were calculated as follows: FIC of ravuconazole = 50% effective concentration (EC_50_) of ravuconazole in combination/EC_50_ of ravuconazole alone (the same equation was applied to benznidazole) and ∑FICs = FIC ravuconazole + FIC benznidazole. An overall mean ∑FIC was calculated for each combination and used to classify the nature of the interaction. Isobolograms were constructed plotting the FIC of benznidazole against ravuconazole. Each curve represents the mean of two independent experiments. Serological data were analyzed using the nonparametric version of Tukey's multiple-comparison test. Differences were considered significant if the *P* value was less than or equal to 0.05.

### Ethics.

All procedures and experimental protocols were conducted in accordance with the COBEA (Brazilian School of Animal Experimentation) guidelines for the use of animals in research and approved by the Ethics Committee in Animal Research at UFOP (protocol number 2011/79).

## Supplementary Material

Supplemental material
