# Supplementary material for: Outcome of E1224-Benznidazole Combination Treatment for Infection with a Multidrug-Resistant Trypanosoma cruzi Strain in Mice
Source: Antimicrob Agents Chemother. 2018 May 25;62(6):e00401-18. doi: 10.1128/AAC.00401-18 (PMC5971593; doi:10.1128/AAC.00401-18)
Supplement: Supplemental material [file supp_62_6_e00401-18__index.html]

Supplemental material 

# Outcome of E1224-Benznidazole Combination Treatment for Infection with a Multidrug-Resistant Trypanosoma cruzi Strain in Mice

## Supplemental material

- Supplemental file 1 -

  Supplemental Figure S1

  PDF, 91K
