# Supplementary material for: Outcome of E1224-Benznidazole Combination Treatment for Infection with a Multidrug-Resistant Trypanosoma cruzi Strain in Mice
Source: Antimicrob Agents Chemother. 2018 May 25;62(6):e00401-18. doi: 10.1128/AAC.00401-18 (PMC5971593; doi:10.1128/AAC.00401-18)
Supplement: Supplemental material [file AAC.00401-18_zac006187176s1.pdf]

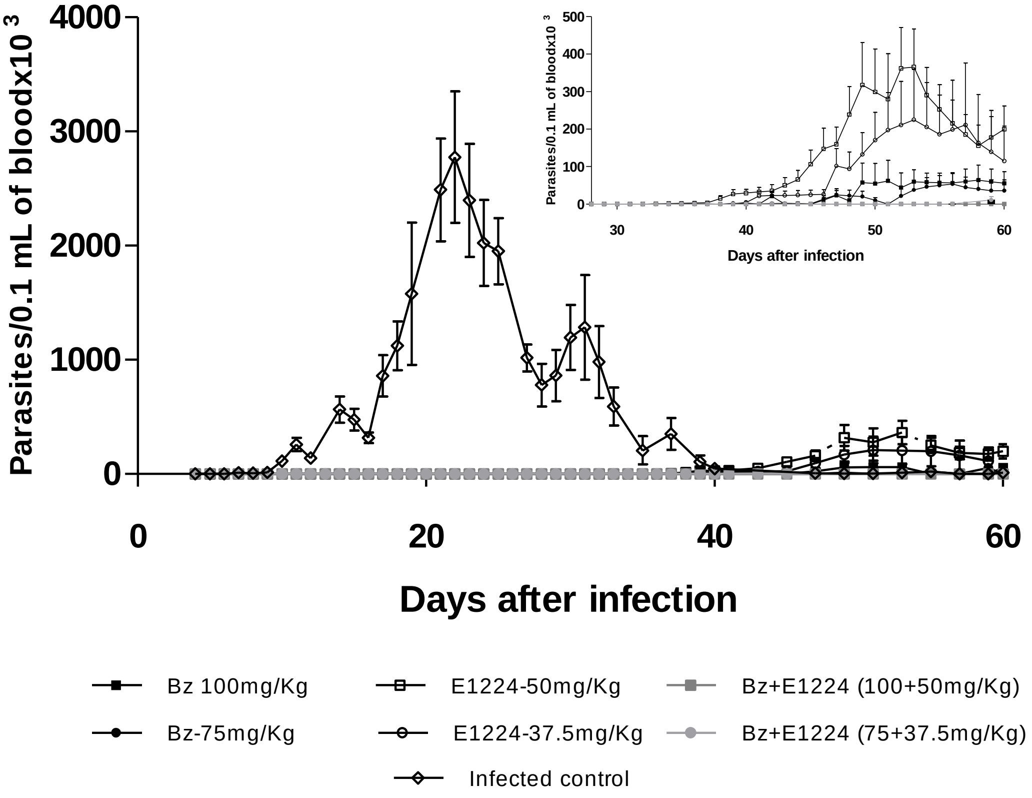

Figure S1 – Parasitemia levels after oral administration of E1224 or benznidazole in monotherapy or combination. Parasitemia curve obtained from mice infected with 5000 trypomastigotes of *T. cruzi* Colombian strain and treated daily with 50 or 37.5 mg/kg of E1224 and 100 or 75 mg/kg of benznidazole alone or in combination for 20 consecutive days. Treatments were started at day 4 of infection. The graph inset shows parasitemia curve only of the treated groups after the treatment period.
